# Social and content aware One-Class recommendation of papers in scientific social networks

**DOI:** 10.1371/journal.pone.0181380

**Published:** 2017-08-03

**Authors:** Gang Wang, XiRan He, Carolyne Isigi Ishuga

**Affiliations:** 1 School of Management, Hefei University of Technology, Hefei, Anhui, People’s Republic of China; 2 Department of Management Science, Kenyatta University, Nairobi, Kenya; Dalian University of Technology, CHINA

## Abstract

With the rapid development of information technology, scientific social networks (SSNs) have become the fastest and most convenient way for researchers to communicate with each other. Many published papers are shared via SSNs every day, resulting in the problem of information overload. How to appropriately recommend personalized and highly valuable papers for researchers is becoming more urgent. However, when recommending papers in SSNs, only a small amount of positive instances are available, leaving a vast amount of unlabelled data, in which negative instances and potential unseen positive instances are mixed together, which naturally belongs to One-Class Collaborative Filtering (OCCF) problem. Therefore, considering the extreme data imbalance and data sparsity of this OCCF problem, a hybrid approach of Social and Content aware One-class Recommendation of Papers in SSNs, termed SCORP, is proposed in this study. Unlike previous approaches recommended to address the OCCF problem, social information, which has been proved playing a significant role in performing recommendations in many domains, is applied in both the profiling of content-based filtering and the collaborative filtering to achieve superior recommendations. To verify the effectiveness of the proposed SCORP approach, a real-life dataset from CiteULike was employed. The experimental results demonstrate that the proposed approach is superior to all of the compared approaches, thus providing a more effective method for recommending papers in SSNs.

## Introduction

The rapid development of information technologies, especially Web 2.0 technology, has changed Internet users from being passive and consumption-driven to being active and production-driven [1]. A variety of platforms that have resulted from Web 2.0 have lifted the barrier of adding information to the Internet and enabled collaborative content creation and modification among users. Among them, social networks, as a typical application of Web 2.0, have become one of the fastest-growing online information interaction platforms in recent years. With the rapid expansion of social networks, social networks specific to the research domain have also appeared. These are referred to as scientific social networks (SSNs), and examples include ResearchGate, CiteULike, Academia.edu, and ScholarMate [2, 3]. SSNs soften research boundaries, strengthen social networking research, and allow researchers to easily find, use, and share research papers. These activities are of great benefit to researchers because they can use them to keep abreast of the current trends in their research fields [2]. However, the rapid increase in the rate at which new papers are published and the ease of sharing them via SSNs have also led to an information overload problem [4, 5]. This makes it difficult for researchers to find the most suitable and interesting scientific papers. From this point of view, building recommendation systems to reduce irrelevant content and provide researchers with the most pertinent papers is an advisable method for helping researchers to relieve the burden of time wasted on irrelevant papers.

Recently, paper recommendation methods have attracted many researchers. Different paper recommendation approaches to automatically find papers from an overwhelming number of available options have been developed [6–11]. A typical approach, content-based filtering (CBF), has roots in digital libraries. It assists knowledge workers with finding helpful documents from the Internet due to its content-oriented nature [12]. CBF analyses the content of a paper based on a researcher’s past behaviour, such as the browsed papers, to extract personal preferences and then recommends papers that are similar to those in which the researcher has shown interest in the past [7, 13]. However, most of the existing CBF techniques emphasized the content of a paper. As a result, problems of inaccurate profile extraction, a lack of recommendation diversity etc. are encountered. These problems highly limit the performance of CBF recommendation systems.

To overcome the above problems, in recent years, another recommendation approach, commonly known as collaborative filtering (CF), has attracted more attention. Given its wide application in e-commerce systems, such as Amazon and Netflix, there have also emerged a large number of explorations in applying this approach in scientific paper recommendation. When the CF approach is being used to recommend a paper, it considers the opinions (in the form of historical preference behaviours) of other like-minded researchers [14]. CF predicts based on the assumption that if some researchers agree on the quality of some papers, they are likely to agree on the quality of other unknown papers. However, in spite of the success and popularity, CF also encounters several limitations that make it unable to achieve the expected performance, including data sparsity and the cold start problem. Of them, data sparsity is a major problem that results from the fact that researchers are not willing to invest effort and time in rating most papers in the real world; hence, the user-item matrix is sparse.

To alleviate the respective disadvantages of the CBF and CF approaches mentioned previously and leverage the advantages of them at the same time, in recent years, hybrid recommendation approaches, in which the CBF and CF are combined, has been proposed to recommend scientific papers [9, 15, 16]. Because of its higher prediction accuracy than a single approach and the disadvantages of one approach can be overcome by the other approach, the hybrid approach has already been gradually replacing the CBF and CF as a research hotspot and the mainstream model.

Unfortunately, in spite of the success and popularity of the hybrid approach in scientific research, most of its existing approaches have demonstrated to not be very robust when used to recommend papers in reality. The reason is that unlike ratings given in the form of explicit numerical ratings, e.g., a1–5scale, to represent the degree tendency from “dislike” to “love”, in SSNs recommendation, there are only positive instances, such as browsing activities and collecting activities that show the user preferences (interested) explicitly, whereas the negative instances of the user (uninterested) are extremely lacking. The extreme imbalance between the positive and negative instances and the extreme sparsity in the dataset make it difficult to train the model and not possible to achieve the expected performance. Formally, the type of data generated from paper recommendation is such that only positive instances can be clearly distinguished, whereas negative instances are highly uncertain; therefore, the problem is a classic One-class Collaborative Filtering (OCCF) problem. However, to the best of our knowledge, employing the perspective of OCCF to study paper recommendation remains has rarely been done.

In an OCCF problem, the data usually consist simply of binary data reflecting a user’s action or inaction, such as page visitation in the case of news recommendation [17]. These ‘action’ items explicitly express the interested tendency of users; therefore, they are observed as positive instances. In contrast, the negative instances are these items that users are not interested in. However, in situations of OCCF, there is only a small part of data that has been labelled positive instances, whereas the rest is all unlabelled, i.e., ‘inaction’ or ‘missing’; thus, the data are usually extremely imbalanced and sparse. In other words, since ‘inaction’ items contain not only items that are not really interested to users but also items in which users are interested but that cannot be found, they cannot be viewed simply as negative instances. According to the earlier mentioned problems, the prior studies about OCCF mainly focused on the modelling of these unlabelled instances [17–19]. However, the information about users in these studies has been restricted to static statistics and transaction information on items only, such as browsing and review activities, which is inadequate and inevitably results in a large amount of noise accompanied by the negative instance introduction. In many applications, we naturally have much social information that can be leveraged. For example, in SSNs, researchers are allowed to assign tags to papers freely. These tags describe the contents of a paper; thus, the latent features of the paper are obviously affected by the tags with which it is labelled. Tags are also provided by researchers themselves independently, so they can more reflect each researcher’s own concerns. Therefore, when building profiles to express users’ preference and papers’ features for similarity calculating and further negative instance extraction, considering this social tag information makes the extraction of researcher preferences and paper features more characterized. On the other hand, with the deepening of communication among researchers in SSNs, many friend relationships have been established by researchers with similar interests, hobbies, and research field independently. Similar to the strength of social tag information used in the procedure of profiling and modelling, this social friend information also plays an important role in paper recommendation because people tend to be more willing to choose items that have been chosen by their friends in real world [4]. That is, the preference of a specific researcher should be similar to that of his friends to some extent. Nevertheless, despite these benefits of this rich social information, in the existing setting of recommending for OCCF problem, there has been little exploration of how to mine the social information in SSNs to overcome the imbalance and sparsity problem of the data and improve the recommendation performance.

Therefore, to address the specific problems noted above and further improve the performance of OCCF, a hybrid approach of Social and Content aware One-class Recommendation of Papers (SCORP) in SSNs is proposed in this study. First, when extracting negative instances to alleviate the problem of imbalance and sparsity, in addition to explicit positive instances of researchers’ historical behaviours, the social tag information is applied into the traditional CBF to profile the researcher preferences and paper features, respectively. Since these social tags are the description of papers’ main contents provided by researchers themselves subjectively, they express not only the features of papers but also the concerns of researchers. Therefore, using them to calculate the similarity between researchers and their unread papers as a probability of the paper to be extracted as a negative instance is more trustworthy. Then, based on the similarities, the unlabelled papers of high dissimilarity with that target researcher are extracted as negative instances. Additionally, the more positive instances the researcher has acted in the past, the higher his activity, and consequently, the more the negative instances are extracted for him. This is because the more positive instances the researcher has acted, indicating that the more papers he has seen previously. Other papers on which he has not acted are more likely to be seen, but not be interesting to him rather than not be seen; therefore, the more negative instances should be extracted for him. Second, in the step of modelling and predicting, the social information in SSNs, such as tags and friends, is embedded into the standard CF approach. In the real-world scenarios of OCCF, a user typically interacts with a limited number of items out of possibly thousands or millions of items, which leads to extremely sparse user implicit feedback. It should be noted that although the negative instances are first extracted from the missing data to enlarge the size of the labelled instances, the extracted number of negative instances is determined by the number of positive instances and must not be excessive. Excessive negative instances extraction will inevitably bring a large amount of noise, resulting in another imbalance, that is, too many negative instances and too few positive instances. An extreme is all missing as negative, of which all missing potential positive instances are extracted as negative instances mistakenly. It is obviously not worthwhile to fill the data and consequently sacrifice recommendation performance. Moreover, on the one hand, as mentioned above, the social tag information in SSNs is a visually textual description of the papers’ contents. Therefore, the latent feature of the paper is obviously affected by the tags with which it has been labelled. On the other hand, in reality, people always turn to their friends for recommendations; naturally, their tastes and characters are easily affected by the friends they keep. In view of the abovementioned reasons, the social information in SSNs is incorporated into the traditional CF approach as additional information to further relieve the effect of data sparsity in OCCF. The proposed hybrid SCORP was evaluated through the comprehensive experiments using real-life data from CiteULike, a leading scientific social network. The experimental results demonstrate that the proposed SCORP approach achieves its best performance, an F-measure of 0.03341 when recommending 10 papers and an MAP of 0.09035 when recommending 20 papers. Moreover, compared with the baseline approaches, the proposed hybrid SCORP can yield an improvement of more than 19% in terms of the F-measure, thus providing an effective method to recommend papers in SSNs.

The main contributions of this paper can be summarized as follows:

1. A hybrid approach of Social and Content aware One-class Recommendation of Papers, termed SCORP, in which the recommendation of paper is formalized as an OCCF problem, is proposed to recommend papers in SSNs. To the best of our knowledge, this problem has rarely been explored in the state-of-the-art paper recommendation studies regarding SSNs.
2. To solve the extreme imbalance problem, the social information in SSNs is applied in the CBF approach for profiling to determine which papers are more likely to be selected as negative instances for a target researcher.
3. To solve the extreme sparsity problem, when modelling and predicting, social information, such as tags and friends, is integrated into the standard CF approach to enhance the quality of it.
4. To evaluate the performance of the proposed hybrid SCORP and the impacts of the integrated social information, comprehensive experiments were conducted using a real-life dataset, CiteULike. The experimental results demonstrate the effectiveness of the proposed approach.

The rest of the paper is organized as follows. In the following section, we survey the related work about paper recommendation and OCCF. The details of the hybrid SCORP approach are introduced in the Hybrid SCORP approach in scientific social networks section, whereas the Experimental design section presents the dataset, metrics and compared approaches used in the experiments. The results are analysed in the Results and discussion section. We draw conclusions in the Conclusions and future work section and discuss future research.

## Related work

Our work is related to two main research threads: (1) paper recommendation and (2) OCCF. We review the pertinent existing literature from these two fields.

### Paper recommendation

Paper recommendation systems, as mentioned earlier, are systems capable of helping researchers find papers that are relevant to their interests. They can be classified into three categories: content based filtering systems (CBF) [20], collaborative filtering systems (CF) [21–23] and hybrid systems [9, 24–26]. CBF identifies items of special interest through analysing item descriptions, whereas CF filters or evaluates items by users’ opinions. Hybrid systems combine CBF and CF to improve the accuracy of recommendations. Details about the current state of paper recommendation already performed by researchers are given below.

The CBF approach tries to retrieve useful information, which is usually textual data from papers that the researchers have shown interest in the past to build researcher preference profiles. Then, relying on the researcher’s behavioural history together with the paper feature profiles, a list of new papers that are similar to the researcher preference are recommended [9]. Case studies of typical CBF recommendation approaches were recently presented by Nallapatiet al. [27], in which the text and citation relationship were jointly modelled under the framework of topic model. For the effective performance of the CBF, personal preferences are required to be identified for the researchers and papers [7]. Basu et al. [28] proposed a technical paper recommendation approach in which multiple information sources were combined to represent the researchers’ preferences and the papers’ features. A personalized research paper recommendation systems was introduced by Honget al. [29], in which a user-profile-based algorithm was used to extracting keyword by keyword extraction and keyword inference. Besides, recommendation system presented in [13] was based on similarities between researcher profiles and the concepts of each paper in the background data. In [4], a scholarly paper recommendation system was developed by Nascimento et al.; they used titles to construct researcher profiles and titles and abstracts to generate feature vectors of candidate papers to recommend. However, most of the above pioneering CBF fails to represent the papers accurately because it suffers the limitation of content feature extraction. With limited features, there will be the situation that several different items are represented to the same features. It is obvious difficult for the CBF to distinguish them. Another problem of employing the CBF is the lack of recommendation diversity. In other words, it cannot recommend papers that are different from those a user liked in the past. This is contrary to the goal of recommendation systems, which is to provide users with a wide range of options, not homogeneous set of alternatives [9]. In addition to these problems, new researchers and ignorance of the opinions of other researchers are all problems arising in the CBF.

With the prevalence of social bookmarking and social networking websites, the CF approach has attracted increasingly much attention. It recommends papers for researchers based on the information on the likes or dislikes of a researcher. For example, in SSNs, given a target researcher’s ratings for several papers and a database of other researchers’ ratings, the CF predicts how the target researcher would rate a paper that he has not read. The key idea of CF is that a target researcher prefers those papers that like-minded researchers preferred. For example, Boger and Bosch [2] applied the traditional CF approach for recommending scientific papers and found that the user-based approach was better than the item-based approach because of the data distribution factors. The experimental results showed that owing to tag contribution, BM25-boosted CF achieved better performance than the other two CF approaches used, Classic CF and Neighbour-weighted CF. To overcome the data sparsity problem, Vellino [30] proposed a hybrid multi-dimensional approach in which usage-based and citation-based approaches for recommending research papers were combined. A similar approach was also presented in [6], which provided an interpretable latent structure with CF and probabilistic topic modelling for researchers and papers; this approach is thus able to make recommendations for both existing and newly published papers. In addition to collective relations involved in user-item pairs, some attempts have been made to analyse and incorporate social relations into the CF approach. Lee and Brusilovsky [31] conducted paper recommendation considering self-defined social contacts; group trust was mined together with personal trust and incorporated into the traditional CF techniques to recommend papers. In [32], a social citation network was used in the CF approach to build a citation graph of papers for recommendation. It is clear from these studies that the CF approach, as a popular and successful approach in e-commerce domain, can also be used for paper recommendation and can provide recommendations that are unable to be considered by the CBF [33–35]. However, the CF approach still have some potential limitations, such as sparsity and cold start issues [36, 37]. In particular, the sparsity issue entails low-quality recommendation results being obtained because the system has very few rating records that users can employ to measure the similarity between users or items. These data sparsity and cold start problems are non-negligible problems with employing CF for paper recommendation that must be addressed.

In hybrid recommendation approaches, the CBF and CF approaches are combined to exploit synergy between them [15]. The main assumption for this approach can be stated that “combining the approaches can provide more accurate recommendation than a single approach and disadvantages of each approach can be overcome by the other approach” [38]. The recommendation system in [39] employed a two-stage hybrid approach. First, it adapted a CBF by representing books with keyword features to find neighbours. Then, it generated a CF-based recommendation list and removed irrelevant books from the CF-based list using the keyword preferences of individual members. Kodakateri [40] reversed the stages. This method executed CBF based on the result trained through the decision tree, instead of incorporating the CBF approach into the CF approach. In [41], another hybrid approach was proposed by switching between the co-authorship network-based and content-based techniques on the basis of the content coherence of a task profile. The abovementioned studies have combined CBF and CF approaches to exploit the benefits of each other and lessen their disadvantages. Many experiments with different combined strategies have been conducted in such studies, and these experiments have proved the effectiveness of hybrid approaches. It is important to note that in SSNs, there are only a few positive instances that can be observed explicitly, leaving a vast number of unlabelled instances mixed with negative and unseen papers, leading to extreme imbalance and sparsity; this is thus a classic OCCF problem. Nevertheless, few of approaches have considered the OCCF perspective.

### OCCF

The OCCF problem, proposed in [17], is the problem of learning from positive and unlabelled instances. It can be said that negative instances and unseen potential positive instances are mixed together, and usually, the portion of positive instances is small, which leads to an extreme data imbalance and data sparsity. Recently, one-class datasets have started to obtain the attention of researchers, and some relevant research addressing the aforementioned two limitations has been performed. These studies are mainly based on one of two main lines of thought.

The first line is at the algorithm level. For example, Pan et al. [17] proposed a weighted low-rank approximation in which cost-sensitive learning is used. The central idea is to treat all missing values as negative instances but give low and different weights to the error terms of negative instances in the objective function. Moreover, in addition to assign a uniform weight ∈[0, 1] for the credibility of each missing data being a negative instance over all users or all items, there were two other weighting schemes that have been discussed, namely, user-oriented and item-oriented. Specifically, they think if a user has viewed more items, those items that he has not viewed are more likely to be negative; if an item is viewed by fewer users, the missing data for this item are more likely to be negative. Accordingly, after computing the number of items each user has rated and the number of users each item has been rated by, the weights were determined. In a subsequent paper by Hu et al. [42], vastly varying confidence weights on implicit feedback were introduced, which can then be approximated into a scalable optimization procedure by two latent feature matrices. However, both of them require auxiliary knowledge of confidence for each observed feedback, which may not be available in real applications and cannot be easily transferred to other situations. Furthermore, the strategy of weight determination is always a restriction that cannot be ignored, but as far as we know, a useful solution of this has not yet been proposed. Different from making a uniform assumption about the negative class that are exploited in the above studies, Sindhwani et al. [19] suggested treating zero-valued pairs as optimization variables computed from the training data. Therefore, the distribution of the negative class was learned. Rather than these approaches of formulating an optimization problem, Paquet and Koenigste [43] addressed the lack of a negative class as a probabilistic model using a Bayesian generative model for the latent signal with an unobserved random graph that connects users with items that they might have considered. However, all of the above studies are based on the strategy of treating all missing values as negative instances, thus resulting in low practicability and applicability owing their high computational costs, especially for large-scale sparse datasets, which are common in real recommendation scenarios. Recently, more studies have focused on how to take advantage of the abundant additional information to treat data imbalance and data sparsity. A latent factor model was proposed to collaboratively mine users’ brand preferences across multiple domains simultaneously [44]. Via collective learning, the learning processes in all domains are mutually enhanced; hence, the problem of data sparsity in every single domain can be effectively addressed by using information of other domains to a certain extent. In [45], unlike the social interactive friend information that is exploited in our study, static historical behaviours, including a user’s search query history, and purchasing and browsing activities, were incorporated to improve the OCCF accuracy. Experimental results have demonstrated the validity of these models.

Another line of OCCF is at the data level. The idea is to use sampling to re-balance the data and alleviate the data sparsity. In prior works, several intuitive strategies to address this problem have been presented. One common solution is to treat all the missing data as negative (AMAN). Empirically, this solution works well (see Section Experimental results and analysis). The drawback is that it biases the recommendation results because some of the missing data might be positive. Another solution is to treat all the missing data as unknown (AMAU), which ignores all missing instances and only utilizes positive instances in the models. This was performed in the one-class SVM [46], which tries to learn the support of the positive distribution. A trivial solution arising from this approach is that all the predictions for missing values are positive instances. Obviously, this prediction is unreasonable. AMAN and AMAU are two extreme strategies in OCCF. Recently, a related family of studies focused on how to tune the trade-off between AMAN and AMAU, i.e., the induction of truthful and moderate negative instances, and consequently develop better-performing algorithms overall. Such a strategy was first proposed by [17], in which, a fast random sampling algorithm was used to introduce negative instances from unlabelled data based on a probability matrix and negative instance size. Specifically, the probability matrix was generated on the basis of three schemes, uniform random sampling, user-oriented sampling and item-oriented random sampling. The assumptions behind them are the same as the weight determination schemes that we explained above. Following this research direction, more sophisticated assumptions attempt to balance the solution and improve over the two extreme ones. A further study was conducted to explore the scalability of algorithms [18]. The experimental results for a large-scale dataset demonstrate that low-rank approximations and maximum-margin matrix factorization were truly valuable in practice. Then, in [47], the authors proposed an approach to explicitly address this type of ambiguity by instead treating the unobserved items as optimization variables. These variables are optimized in conjunction with learning a weighted, low-rank nonnegative matrix factorization (NMF) of the user-item matrix as the optimized model in a group matrix factorization. Such frameworks provide a convenient method of combining feedback from preferences and from reviews, but their main drawback is that they are only applicable to review websites and cannot be easily transferred to other situations. Alternatively, in addition to extracting the negative instances from unlabelled data, in the last year or two, there have been new attempts to identify users’ dislike negative instances from their observed ‘action’ feedback. The sentiment of free-form user comments was combined with a nearest neighbours model into a sentiment-aware nearest neighbour model (SANN) by mapping the sentiment scores to user ratings [48]. The problem of OCCF was converted to recommendations with multiple scores with which the degree of like and dislike is clearly distinguished. A similar exploration was conducted in [49], where the sentiment of comments was explored and mined by an ensemble learning-based sentiment classification (ELSC) approach and then integrated with a matrix factorization framework. The benefits of these two approaches were mostly observed when the amount of user comments was sufficient. In cases in which user comments are few or even unavailable, such as paper recommendation in SSNs, their performance degrades.

In general, in the circumstances of solving the extreme data imbalance and data sparsity problems naturally faced in OCCF, as noted above, no matter working at the algorithm or data levels, there are serious limitations. When determining the confidence weight or the probability of the unlabelled data to be a negative instance, only the static transaction statistical properties of users’ historical behaviour are used. As a result, there is no distinction between all missing items for a user or all missing users for an item. Obviously, it is not reasonable and not personalized for each user-item pair, since for different users, the possibility of different missing items being negative instances is different. From this point of view, there is an urgent need to study discrimination of specific items for each user. More importantly, in many applications, we naturally have much social information that can be leveraged as additional information. For example, in SSNs such as CiteULike, researchers can assign tags to papers they have read and establish relationships with people of their choosing. Therefore, a large amount of observed social information related to the researchers’ preferences and the papers’ features is obtained. This social information, which has been applied in other domains, has been demonstrated to significantly enhance the recommendation quality [50–53]. However, to the best of our knowledge, in the setting of OCCF for paper recommendation in SSNs, how to exploit this social information to overcome the data imbalance and data sparsity problems has been rarely explored. Therefore, in this study, a hybrid SCORP approach in which the social information is mined into the CBF for negative examples extraction and the CF for recommendation modelling is proposed.

## Hybrid SCORP approach in scientific social networks

Scientific social networks are mostly research-centred community platforms with high degrees of paper sharing and communicating. On these platforms, researchers can assign tags to the papers that they have read and build friend relations with other researchers who share similar interests, hobbies or research fields with them. Moreover, the researchers’ actions on papers in SSNs, such as marking as collecting, can only express positive ‘interest’ in papers. However, it is generally not possible to know papers that are not of interest to researchers. Having considered these characteristics of SSNs, a hybrid method, SCORP, is proposed to help researchers find useful papers for their research.

**Fig 1:**
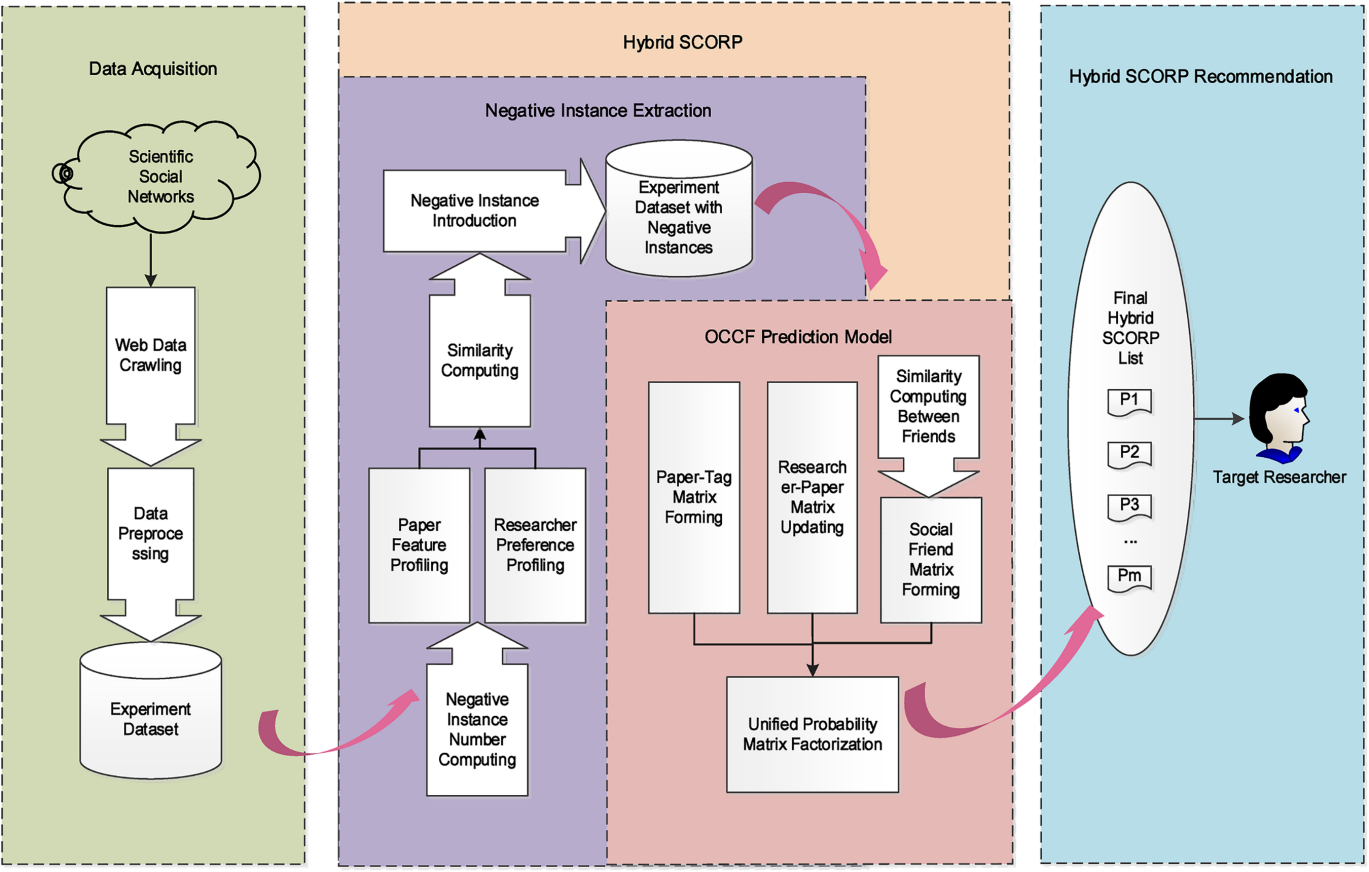
Overview of the hybrid SCORP.

In this section, we will introduce the details of our hybrid SCORP recommendation model. First, we formally define the problem of paper recommendation. Suppose that we have *M* researchers, with the *i*-th researcher denoted as *u*_*i*_, and *N* papers, with the *j*-th paper denoted as *v*_*j*_. Let *R* = {*R*_*i*,*j*_} denote the *M* × *N* researcher-paper matrix, where *R*_*i*,*j*_ represents the action a researcher has performed on a paper. For a pair of vertices *u*_*i*_ and *v*_*j*_, let *R*_*i*,*j*_ = 1,1 ≤ *i* ≤ *M*,1 ≤ *j* ≤ *N* denote that the researcher *u*_*i*_ has performed an action, such as browsing or collecting, on the paper *v*_*j*_ and *R*_*i*,*j*_ = ∅ otherwise. Then, the goal of our hybrid SCORP approach is converted to exploit the observed one-class feedback and predicting the unobserved entries in the researcher-paper matrix *R*^*M*×*N*^ based on the observed entries and other social information. The framework of the proposed hybrid SCORP approach contains three primary stages: data acquisition, negative instance extraction, and an OCCF recommendation model. First, negative instance extraction is employed to extract negative instances for every researcher *u*_*i*_ personalized according to the activity and social tag information of the researcher. Subsequently, based on the researcher-paper matrix *R*_*i*,*j*_ with negative instances extraction completing, social information is embedded into the standard CF approach to further conduct a hybrid recommendation. Fig 1 shows an overview of the proposed hybrid SCORP approach.

### Data acquisition

A web crawler is used to search the Web and retrieved all papers, researchers and friend relations in a certain SSNs platform. For the papers, each piece of information that may be used to represent its features, such as title and tag, are collected together with the browsed and collected histories of researchers. Therefore, a full technical datasheet for each paper is obtained. For each researcher, in addition to his personal basic information and the tags that he has given to papers, the friend relations that they have built with other researchers are collected.

The collected data are analysed and pre-processed to generate the researcher and paper vectors. The data pre-processing consists of the following basic process: filtering, cleaning, tokenization, term extraction and stemming [54]. We first merge duplicate papers, remove empty papers, and remove researchers with too few papers to filter the data overall. Then, numbers, special characters, and punctuation are removed from the data by performing a cleaning task. Next, using Lucene, a full-text search engine toolkit, the tokenization and the term extraction process is conducted [55]. For each paper contained in the data collection, after tokenizing all of their text contents, such as titles and abstracts, into individual terms, stop words are subsequently removed from the data via term extraction. Stop words include non-informative terms that are not proper for representing the domain and the definite article. For example, the words that appears in nearly all papers, such as ‘the’ or ‘a’. Finally, the stemming process considers different terms having the same root as the same terms. The purpose of the data pre-processing is to transform unstructured text data into a structured form represented by words to extract meaningful knowledge.

### Negative instance extraction with social information

As mentioned earlier, when applying the OCCF for paper recommendation in SSNs, the extreme imbalance and sparsity of data greatly reduce the performance of recommendation systems. Therefore, it is an important task to consider how to extract a proper number of negative instances from the papers that the researcher has not taken action on. However, most of the existing studies are only based on the static statistical information to randomly extract negative instances. That is, for each user, all the items that he has not acted on are of the same possibility to be selected as a negative instance, or, for each item, all the users who have not acted on it have the same probability of regarding it as a negative instance [17]. Apparently, the possibility of different inaction items as negative instances is different. In addition, it is known that in SSNs, researchers are able to assign tags to the papers that they have read. Such available social tag information, as a type of implicit feedback, can be fully exploited and combined with the observable positive feedback to improve the recommendation performance. Unfortunately, this has always been ignored in OCCF paper recommendation. Therefore, in view of the above problems, a negative instance extraction approach with the activity and social information is proposed to solve the extreme imbalance and sparsity problems inherent in traditional CBF.

In sum, the main tasks of negative instance extraction include negative instance number computing, paper feature profiling, researcher preference profiling, similarity computing and negative instance introduction. First, for each researcher *u*_*i*_, according to the degree of his activity, the number of negative instance to be selected is determined as follows:

$$N_{i}=\beta \times \sum_{}^{}R_{i}$$ (1)

Where $\sum_{}^{}R_{i}$ is the total number of papers that the researcher *u*_*i*_ has acted on and *β* is a negative rate parameter to control the balance between the positive and negative instances. This parameter is used to express the activity of that researcher *u*_*i*_ based on the assumption that the more active a researcher, the more papers he has already seen previously. Therefore, others that have not been acted are more likely to be seen, but not liked by the researcher, rather than not have been seen. For researcher with high activity, there is a great necessary to extract more negative instances.

Second, for each paper *v*_*j*_, the paper feature profiles are built. The paper feature profile contains a set of word features that describe and characterize a particular paper *v*_*j*_. A paper profile is a vector that obtained by analysing the textual information contained in that paper, such as the title, and social tags assigned to it. For each word feature contained in a paper profile, the frequency of the word to that specific paper *v*_*j*_ is used to denote the weight of the feature. For example, *paperProfile*(*v*_*j*_) is the profile of a paper *v*_*j*_, and it contains the description of *v*_*j*_. *paperProfile*(*v*_*j*_) can therefore be defined as a vector of weights $\left(w_{v_{j}1},w_{v_{j}2},\ldots ,w_{v_{j}t},\ldots ,w_{v_{j}l}\right),1\leq t\leq l$, where each weight $w_{v_{j}t}$ represents the importance of a word feature *k*_*t*_ to the paper *v*_*j*_.

Third, in a similar manner, the researcher preference profiles are built with the textual information of papers he has read and other social information. Therefore, the preference of a researcher *u*_*i*_ can be expressed. Likewise, *researcherProfile*(*u*_*i*_) can be defined as a vector of weights $\left(w_{u_{i}1},\;w_{u_{i}2},\ldots ,w_{u_{i}s}\ldots ,w_{u_{i}r}\right),1\leq s\leq r$, where each weight $w_{u_{i}s}$ represents the importance of a word preference *k*_*s*_ to the researcher *u*_*i*_.

Fourth, the similarity of the profiles between the target researcher *u*_*i*_ and the papers unread by *u*_*i*_ is computed. With the paper feature and researcher preference profiles already been built, similarity between a set of words from *paperProfile*(*v*_*j*_) and *paperProfile*(*v*_*j*_) is obtained. To determine the papers that are dissimilar to ones a researcher *u*_*i*_ preferred in the past, the cosine similarity measure is used as follows:

$$sim\left(u_{i},v_{j}\right)=\frac{\sum_{t}researcherProfile{\left(u_{i}\right)}_{t}\times paperProfile{\left(v_{j}\right)}_{t}}{\sqrt{\sum_{t}researcherProfile{\left(u_{i}\right)}_{t}{}^{2}}\times \sqrt{\sum_{t}paperProfile{\left(v_{j}\right)}_{t}{}^{2}}}$$ (2)

In this formula, *researcherProfile*(*u*_*i*_)_*t*_ is the t-th component of the researcher preference profile *researcherProfile*(*u*_*i*_), and *paperProfile*(*v*_*j*_)_*t*_ is the t-th component of the paper feature profile *paperProfile*(*v*_*j*_). The similarity values *sim*(*u*_*i*_,*v*_*j*_) range from 0 to 1; the nearer the values to 0, the lower the similarity, which indicates a higher likelihood that researcher *u*_*i*_ dislike the paper *v*_*j*_. This calculation is repeated for all papers that have not been acted on by *u*_*i*_. Then, a paper order is determined based on these calculated results for researcher *u*_*i*_, arranged from small to large.

**Fig 2:**
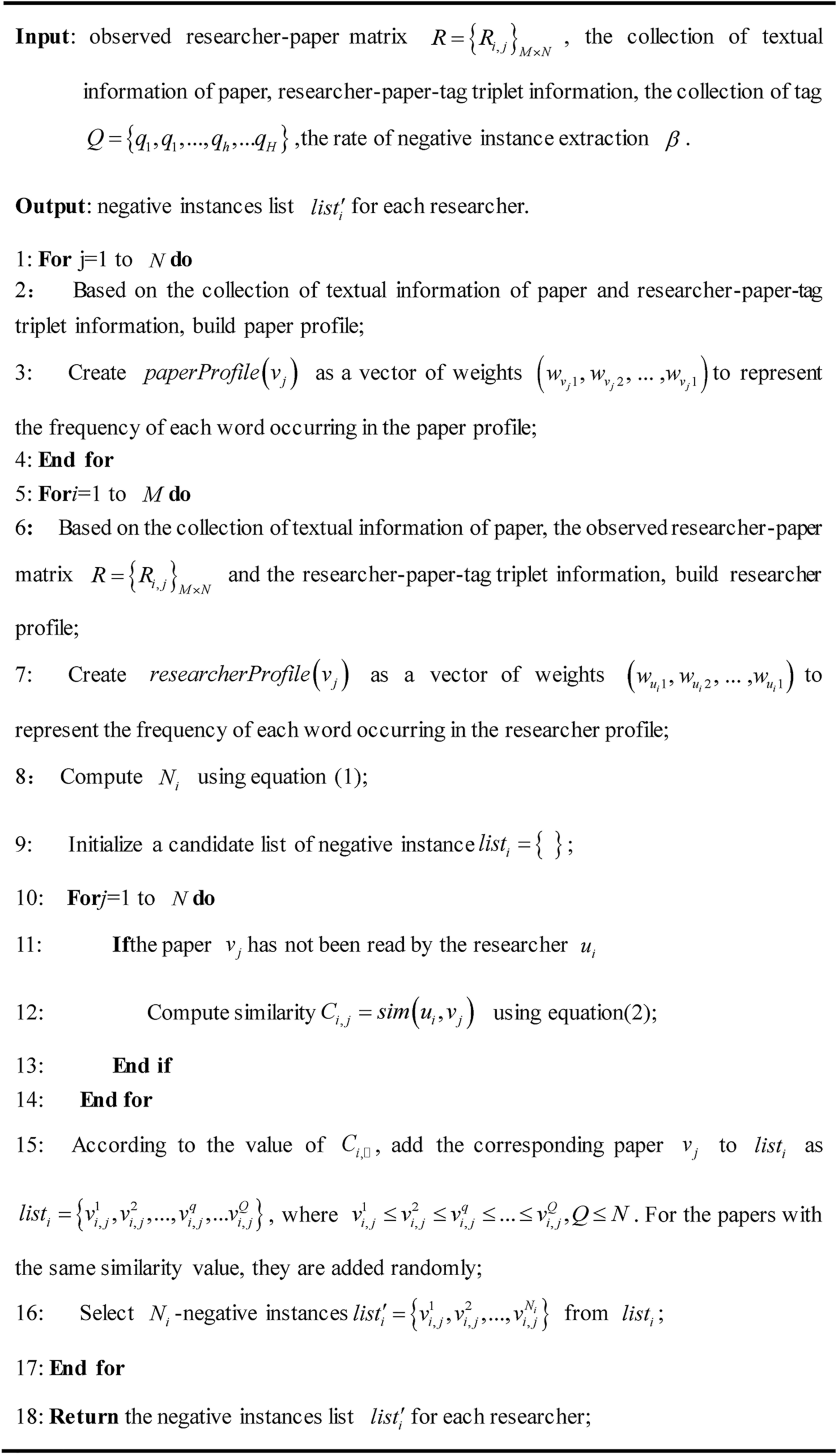
The algorithm of negative instance extraction with social information.

Finally, the negative instances for each researcher are introduced. For a target researcher *u*_*i*_, after all papers on which he has not acted are ranked according to the similarity values *sim*(*u*_*i*_,*v*_*j*_), only the top *N*_*i*_ papers are extracted as negative instances. In the case of having the same similarity, papers are randomly selected. Based on the above analysis, the negative instance extraction of the proposed hybrid SCORP is performed according to the dissimilarity between the researcher preference profiles and paper feature profiles. These papers that have a high degree of dissimilarity with a researcher’s preferences are selected. Fig 2 shows the algorithm of the negative instance extraction process.

### OCCF prediction model with social information

Unlike the CBF approach, the CF approach predicts papers to researchers based on their previously rated papers. In general, CF approaches are mainly divided into memory-based CF and model-based CF [56]. In memory-based CF, the recommendation score is computed based on the entire the user-item matrix. The most common approach to memory-based CF is based on neighbourhood models. The primary task to most of those neighbourhood approaches is the similarity measure between users or items. However, in regard to the extreme imbalance and sparsity problem of OCCF, all these approaches share the disadvantage that they cannot provide an accurate similarity when searching for neighbourhoods [57]. Model-based CF approaches attempt to learn complex patterns based on training data (for example, the user-item rating matrix) and provide recommendations based on the learned models [58]. Matrix factorization (MF) techniques are typical model-based CF approaches and have been successfully applied in movie recommendation context [59]. As its improved variant, recently, Probabilistic Matrix Factorization (PMF) models have gained increasing popularity because they have exhibited outstanding performance in many domains of recommendation problems [50–52]. In particular, they address the problems of high dimensions and sparse instance spaces. The idea behind the PMF approach is to predict the rating by the learned high-quality *D*-dimensional feature representation *U* of users and *D*-dimensional feature representation *V* of items based on analysing the user-item matrix *R*^*M*×*N*^. In this work, we exploit the PMF to the OCCF for paper recommendation in SSNs through a modification with social information.

Note that in real-world scenarios of OCCF, often less than 1% of the interaction between users and items is observable out of possibly thousands or millions of items, which leads to the extreme sparsity. In the first step of negative instance extraction, to avoid introducing noise, the number of negative instances is determined based on the user’s activity. In other words, even if negative instances have been extracted, the data are still sparse compared with the large amount of available data. In addition, in SSNs, as a special form of social networks, there is a large amount of social tag and friend information created by researchers independently. This social information has been proved to significantly improve the recommendation performance [60]. To be specific, this social tag information is the description of paper labelled by the researchers who have read it. Therefore, the latent characteristic matrix of papers is obviously affected by it. In view of the above consideration, both the item-tag matrix and user-item matrix are connected through a shared item latent feature space. That is, the item latent feature space of the item-tag matrix is the same as that of the user-item matrix. Similarly, the latent characteristic matrix of researchers is reasonably expected to be similar with his friends. Since in reality, users normally ask friends for recommendations, their preferences can easily affected by their friends [51]. The higher the similarity between the user and his friends, the closer the latent characteristic matrices of them are [60, 61]. In this study, this social information is embedded into the PMF approach to conduct a unified probabilistic matrix factorization to further alleviate the data sparsity of OCCF for paper recommendation in SSNs.

In general, the OCCF prediction model with social information is implemented in the following five stages: paper-tag matrix forming, similarity computing between friends, social friend matrix forming, researcher-paper matrix updating, and unified probability matrix factorization.

First, the paper-tag matrix is formed. Given the list of tags included in the dataset, let *T* = {*T*_j,*h*_} denotes the *N* × *H* matrix of paper-tag pair information. For each *T*_j,*h*_ in the paper-tag matrix *T*^*N*×*H*^, if the tag *b*_*h*_ has been used to label the paper *v*_*j*_ in the past, *T*_j,*h*_ = 1, and *T*_j,*h*_ = ∅, otherwise.

Then, the similarities for each user with their friends are computed. Considering the reality in SSNs, when searching for papers to browse, the influence of different friends’ recommendation on the researchers must be different. These unimportant noisy friend recommendations have a detrimental effect on the recommendation results. Therefore, it is necessary to measure the degree of similarity to prune this noise. Let *researcherProfile*′(*u*_*i*_) denotes the papers they have read. Please note that unlike a vector of weights defined in the previous section, the $researcherProfile^{\prime }\left(u_{i}\right)=\left\{a_{u_{i}1},a_{u_{i}2},\ldots ,a_{u_{i}g}\ldots ,a_{u_{i}G}\right\},1\leq g\leq G$ here is a list of strings that can uniquely denote a paper, such as the paper ID. Each element $a_{u_{i}g}$ indicates whether the paper it denotes has been acted on by the user *u*_*i*_ at least once. Given a researcher *u*_*i*_ and his friend researcher *u*_*k*_, the similarity, *sim*(*u*_*i*_, *u*_*k*_), between them is obtained by using the Jaccard measure as follows:

$$sim\left(u_{i},\;u_{k}\right)=\frac{\left|researcherProfile^{\prime }\left(u_{i}\right)\cap researcherProfile^{\prime }\left(u_{k}\right)\right|}{\left|researcherProfile^{\prime }\left(u_{i}\right)\cup researcherProfile^{\prime }\left(u_{k}\right)\right|}$$ (3)

The results range from 0 to 1, and a bigger the value of *sim*(*u*_*i*_, *u*_*k*_) indicates more similar between researcher *u*_*i*_ and *u*_*k*_.

Next, the social friend matrix is formed based on the similarities between all researchers and their friends. Let *S* = {*S*_*i*,*k*_}, 1 ≤ *k* ≤ *M* denotes the *M* × *M* matrix of researcher social friend networks. For any two researchers *u*_*i*_ and *u*_*q*_, if they are friends in the SSN, the value of *S*_*i*,*k*_ is the similarity *sim*(*u*_*i*_, *u*_*k*_) of them; otherwise, *S*_*i*,*k*_ = 0.

Subsequently, the researcher-paper matrix is updated with the negative instances extracted for all researchers. To learn the latent features of the researchers and papers, PMF is employed to factorize the researcher-paper matrix. The researcher-paper matrix *R*^*M*×*N*^ is a collection of personal historical interest about all researchers, which is the basis of the PMF approach. To address the key challenges of seriously data imbalance and data sparsity highlighted previously, the negative instances extracted in the previous section are introduced into the initial *R*^*M*×*N*^. Moreover, it is worthwhile to indicate here that instead of assigning -1 to all of the negative instances, the values of them in researcher-paper matrix *R*^*M*×*N*^ are set with the similarity between the negative instances and researchers. This is because in the researcher-paper matrix *R*^*M*×*N*^, *R*_*i*,*j*_ = −1 represents a researcher’s real action (dislike) on a paper, but for the negative instances, they are not the researcher's real selection, so the values of them in *R*^*M*×*N*^ should be close to -1 but never equal -1. Furthermore, based on the assumption that the smaller the similarity of the negative instances with researcher, the more likely they are to be really disliked by that researcher, the similarities are used as the value of the introduced negative instances in *R*^*M*×*N*^.

**Fig 3:**
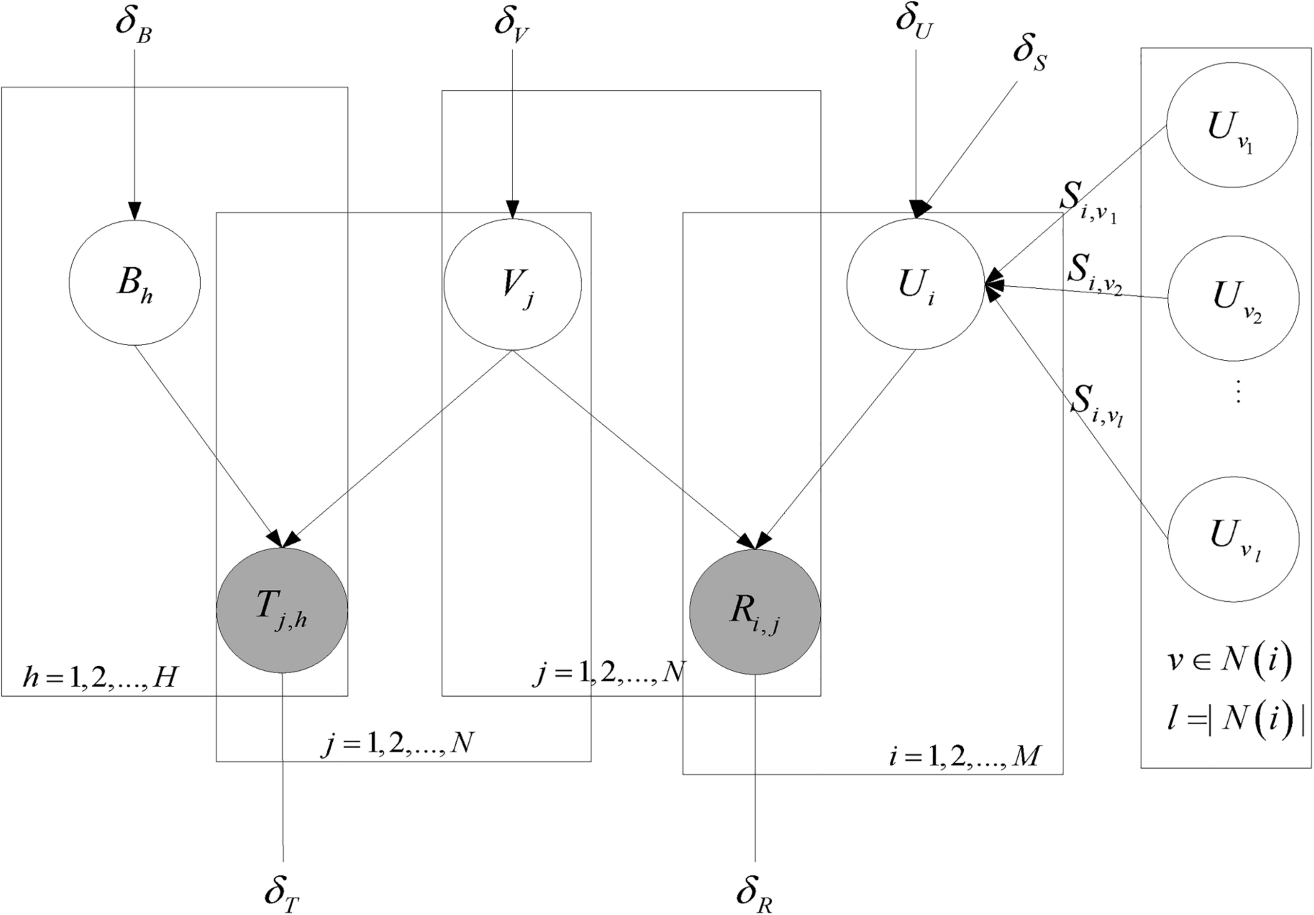
Graph model of prediction with social information.

Finally, unified probabilistic matrix factorization is conducted on the updated researcher-paper matrix *R*^*M*×*N*^ along with the paper-tag matrix *T*^*N*×*H*^ and the social friend matrix *S*^*M*×*M*^. Getting the researchers latent feature matrix *U* ∈ *R*^*D*×*M*^, the papers latent feature matrix *V* ∈ *R*^*D*×*N*^ and the tags latent feature matrix *B* ∈ *T*^*D*×*H*^ through the gradient descent approach. Its probability graph model is shown in Fig 3.

The column vectors *U*_*i*_, *V*_*j*_ and *B*_*h*_ representing the D-dimensional researcher-specific, paper-specific and tag-specific latent feature vector of researcher *u*_*i*_, paper *v*_*j*_ and tag *b*_*h*_, respectively. *S*_*i*,*k*_ indicates the Jaccard similarity between researcher *u*_*i*_ and his friend *u*_*k*_. *N*(*i*) indicates the friend collection of *u*_*i*_. Note that the solutions of *U* and *V* are not unique.

According to the definition above, the conditional distribution over the observed researcher-paper matrix *R*^*M*×*N*^ and the paper-tag matrix *T*^*N*×*H*^ are defined as follows:

$$P\left(R|U,V,\sigma_{R}^{2}\right)={\prod_{i=1}^{M}\prod_{j=1}^{N}\left[N\left(R_{i,j}|g\left(U_{i}^{T}V_{j}\right),\sigma_{R}^{2}\right)\right]}^{I_{i,j}^{R}}$$ (4)

$$P\left(T|V,B,\sigma_{T}^{2}\right)=\sum_{j=1}^{N}\sum_{h=1}^{H}{\left[N\left(T_{j,h}|g(V_{j}^{T}B_{h}),\sigma_{T}^{2}\right)\right]}^{I_{j,h}^{T}}$$ (5)

Where *N*(*x*|*μ*,*σ*^2^) is the probability density function of the Gaussian distribution with mean *μ* and variance *σ*^2^, and $I_{i,j}^{R}$ is an indicator function that is equal to 1 if researcher *u*_*i*_ has read paper *v*_*j*_ (or if paper *v*_*j*_ has been labelled by tag *b*_*h*_), and 0 otherwise. The function *g*(*x*) is the logistic function *g*(*x*) = 1/(1 + *exp*(−*x*)), which makes it possible to bound the range of $U_{i}^{T}V_{j}$ and $V_{j}^{T}B_{h}$ within the range [0, 1].

In addition, to avoid over-fitting, *U*_*i*_, *V*_*j*_ and *B*_h_ are all assumed to subject to zero-mean Gaussian distributions and to be independent of each other. The user feature vector is also affected by their friends' user feature vector at the same time. That is,

$$P\left(U|S,\sigma_{U}^{2},\sigma_{S}^{2}\right)=\prod_{i=1}^{M}N\left(U_{i}|0,\sigma_{U}^{2}I\right)\times \prod_{i=1}^{M}N\left(U_{i}|\sum_{k\in N\left(i\right)}S_{i,k}U_{k},\sigma_{S}^{2}I\right)$$ (6)

$$P\left(V|\sigma_{V}^{2}\right)=\prod_{j=1}^{N}N\left(V_{j}|0,\sigma_{V}^{2}I\right)$$ (7)

$$P\left(B|\sigma_{B}^{2}\right)=\sum_{h=1}^{H}N\left(B_{h}|0,\sigma_{B}^{2}\right)$$ (8)

Hence, through Bayesian inference, we have

$$\begin{array}{l} P\left(U,V,B|R,T,S,\sigma_{R}^{2},\sigma_{U}^{2},\sigma_{V}^{2},\sigma_{T}^{2},\sigma_{B}^{2},\sigma_{S}^{2}\right)\\ \propto p\left(R|U,V,\sigma_{R}^{2}\right)P\left(T|V,B,\sigma_{T}^{2}\right)p\left(U|S,\sigma_{U}^{2},\sigma_{S}^{2}\right)p\left(V|\sigma_{V}^{2}\right)P\left(B|\sigma_{B}^{2}\right)\\ ={\prod_{i=1}^{M}\prod_{j=1}^{N}\left[N\left(R_{i,j}|g\left(U_{i}^{T}V_{j}\right),\sigma_{R}^{2}\right)\right]}^{I_{i,j}^{R}}\times \sum_{j=1}^{N}\sum_{h=1}^{H}{\left[N\left(T_{j,h}|g(V_{j}^{T}B_{h}),\sigma_{T}^{2}\right)\right]}^{I_{j,h}^{T}}\times \\ \;\prod_{i=1}^{M}N\left(U_{i}|0,\sigma_{U}^{2}I\right)\times \prod_{i=1}^{M}N\left(U_{i}|\sum_{v\in N\left(i\right)}S_{i,v}U_{v},\sigma_{S}^{2}I\right)\times \prod_{j=1}^{N}N\left(V_{j}|0,\sigma_{V}^{2}I\right)\times \sum_{h=1}^{H}N\left(B_{h}|0,\sigma_{B}^{2}\right) \end{array}$$ (9)

This equation specifies the approach on how to derive the researchers’ and papers’ latent feature space based on the researcher-paper matrix *R*^*M*×*N*^ with considering the preferences of the researchers’ social friends in addition to the paper-tag matrix *T*^*N*×*H*^. The log of the posterior distribution for the recommendations is given by

$$\begin{array}{l} \ln P\left(U,V,B|R,T,S,\sigma_{U}^{2},\sigma_{V}^{2},\sigma_{B}^{2},\sigma_{R}^{2},\sigma_{T}^{2}\right)\\ =-\frac{1}{2\sigma_{R}^{2}}\sum_{i=1}^{M}\sum_{j=1}^{N}I_{ij}^{R}{\left(R_{i,j}-g\left(U_{i}^{T}V_{j}\right)\right)}^{2}-\frac{1}{2\sigma_{T}^{2}}{\sum_{j}^{N}\sum_{h=1}^{H}I_{j,h}^{T}\left(T_{j,h}-g\left(V_{j}^{T}B_{h}\right)\right)}^{2}-\\ \;\frac{1}{2\sigma_{S}^{2}}\sum_{i=1}^{M}(U_{i}-\sum_{v\in N(i)}T_{i,v}U_{v}{)}^{T}(U_{i}-\sum_{v\in N(i)}T_{i,v}U_{v})-\frac{1}{2\sigma_{U}^{2}}\sum_{i=1}^{M}U_{i}^{T}U_{i}-\frac{1}{2\sigma_{V}^{2}}\sum_{j=1}^{N}V_{j}^{T}V_{j}-\frac{1}{2\sigma_{B}^{2}}\sum_{h=1}^{H}B_{h}^{T}B_{h}-\\ \;\sum_{i=1}^{M}\sum_{j=1}^{N}I_{ij}^{R}\ln\sigma_{R}^{2}-\sum_{j=1}^{N}\sum_{h=1}^{H}I_{j,h}^{T}\ln\sigma_{T}^{2}-D\sum_{i=1}^{M}\ln\sigma_{U}^{2}-D\sum_{j=1}^{N}\ln\sigma_{V}^{2}-D\sum_{h=1}^{H}\ln\sigma_{B}^{2}+P \end{array}$$ (10)

Where *D* denotes the dimension of the feature vector and *P* is a constant that does not depend on the parameters. Maximizing the log-posterior over two latent features with hyper-parameters kept fixed is equivalent to minimizing the following sum-of-squared-errors objective functions with quadratic regularization terms:

$$\begin{array}{l} E\left(U,V,B,R,S,T\right)\\ =\frac{1}{2}\sum_{i=1}^{M}\sum_{j=1}^{N}I_{i,j}^{R}{\left(R_{i,j}-g\left(U_{i}^{T}V_{j}\right)\right)}^{2}+\frac{\theta_{T}}{2}{\sum_{j}^{N}\sum_{h=1}^{H}I_{j,h}^{T}\left(T_{j,h}-g\left(V_{j}^{T}B_{h}\right)\right)}^{2}+\\ \;\frac{\theta_{S}}{2}\sum_{i=1}^{M}(U_{i}-\sum_{v\in N(i)}T_{i,v}U_{v}{)}^{T}(U_{i}-\sum_{v\in N(i)}T_{iv}U_{v})+\frac{\theta_{U}}{2}\sum_{i=1}^{M}U_{i}^{T}U_{i}+\frac{\theta_{V}}{2}\sum_{j=1}^{N}V_{j}^{T}V_{j}+\frac{\theta_{B}}{2}\sum_{h=1}^{H}B_{h}^{T}B_{h} \end{array}$$ (11)

Where $\theta_{u}=\frac{\sigma_{R}^{2}}{\sigma_{u}^{2}}$, $\theta_{v}=\frac{\sigma_{R}^{2}}{\sigma_{v}^{2}}$, $\theta_{T}=\frac{\sigma_{R}^{2}}{\sigma_{T}^{2}}$, $\theta_{s}=\frac{\sigma_{R}^{2}}{\sigma_{s}^{2}}$, $\theta_{B}=\frac{\sigma_{R}^{2}}{\sigma_{B}^{2}}$, which reflect the influence degree of each matrix on the objective function. A local minimum of the objective function given by equal (11) can be found by performing gradient descent in *U*_*i*_, *V*_*j*_, *B*_*h*_:

$$\frac{\partial E}{\partial U_{i}}=\sum_{j=1}^{N}I_{i,j}^{R}\left(g\left(U_{i}^{T}V_{j}\right)-R_{i,j}\right)g^{\prime }\left(U_{i}^{T}V_{j}\right)V_{j}+\theta_{U}U_{i}+\theta_{S}(U_{i}-\sum_{v\in N(i)}S_{i,v}U_{v})-\theta_{S}\sum_{\left\{v|i\in N(v)\right\}}S_{i,v}(U_{v}-\sum_{w\in N(i)}S_{v,w}U_{w})$$ (12)

$$\frac{\partial E}{\partial V_{j}}=\sum_{i=1}^{M}I_{i,j}^{R}\left(g\left(U_{i}^{T}V_{j}\right)-R_{i,j}\right)g^{\prime }\left(U_{i}^{T}V_{j}\right)U_{i}+\theta_{T}\sum_{h=1}^{H}I_{j,h}^{T}\left(g\left(V_{j}^{T}B_{h}\right)-T_{j,h}\right)g^{\prime }\left(V_{j}^{T}B_{h}\right)B_{h}+\theta_{V}V_{j}$$ (13)

$$\frac{\partial E}{\partial B_{h}}=\theta_{T}\sum_{j=1}^{N}I_{j,h}^{T}\left(g\left(V_{j}^{T}B_{h}\right)-T_{j,h}\right)g^{\prime }\left(V_{j}^{T}B_{h}\right)V_{j}+\theta_{B}B_{h}$$ (14)

Where *N*(*k*) is the set that includes all the researchers who are friends of researcher *u*_*k*_ and *g*′(*x*) is the derivative of logistic function *g*′(*x*) = *exp*(*x*)/(1 + *exp*(*x*))^2^.

**Fig 4:**
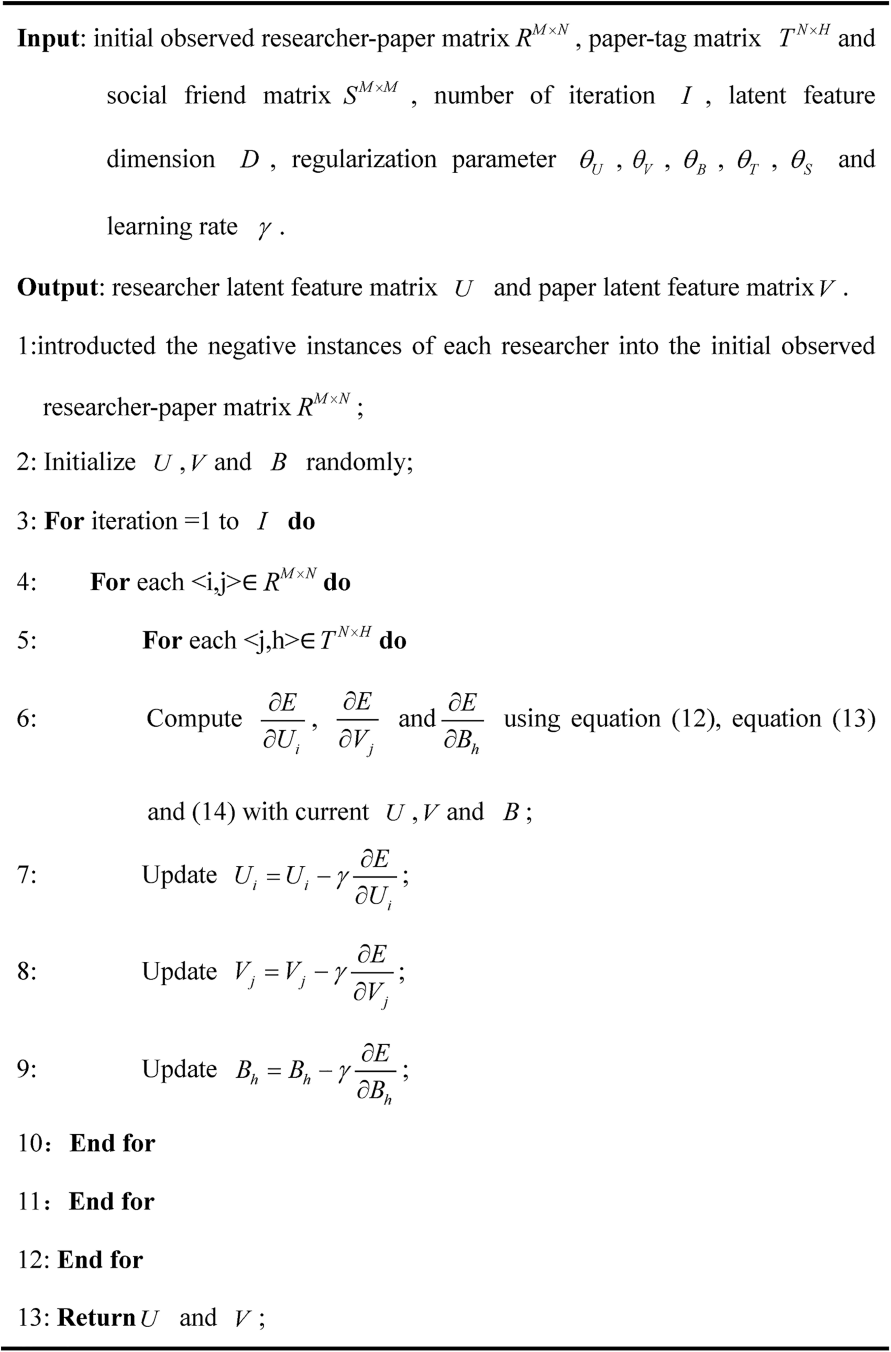
The algorithm of prediction model with social information.

For each researcher, after obtaining all prediction ratings based on the latent characteristic matrix *U* and *V*, the top *N* papers that he has not read are selected. For papers with the same rating, they are selected randomly. This approach predicts how the target researcher would rate a paper that he has not read according to their previously rated papers. Therefore, the higher the rating obtained by a paper, the more likely the target researcher is interested in it. Fig 4 shows the algorithm of the OCCF prediction model with social information.

## Experimental design

This section presents the experiments of the proposed hybrid SCORP. The first two parts present the experimental datasets and evaluation metrics that were used for evaluating the proposed approach. Then, the compared approaches and experimental procedure are explained in detail in the last part.

### Experimental dataset

To evaluate the proposed hybrid SCORP, the CiteULike dataset was investigated in this research [3]. CiteULike is one of the leading SSNs with an established social tagging and friend-making system for researchers to find, store, manage, and share academic papers online. On the website, researchers can add scientific papers in which they are interested to their libraries and use tags to easily comment on any existing papers. Moreover, CiteULike allows researchers to establish friend relationship with anyone they want and communicate scientific research by creating research groups or join others’ groups with specific research topics. Therefore, the CiteULike dataset is suitable for the experimental study performed here. To obtain the required data, the CiteULike website was visited using a crawler. A collection that contains paper Ids and other related information was extracted for each paper. Similarly, for each researcher, his researcher ID was collected together with his browsing history. Since we employed social information to improve the recommendation quality, in addition to papers’ and researchers’ social tags information, the friend relations that were shown on the page at the time of the visit were collected. Considering the incomplete data problem and the comparability of experiment, the tags were mainly used as text description information for the researchers and the papers. Then, a data cleaning task was conducted following the process described in [62]. Papers with less than 5 researchers who have read on them and researchers with less than 15 papers browsing records were removed from the dataset. Finally, a dataset containing 1,024 researchers, 11,375 papers, 78,188 researcher-papers pairs, and 310,301 tags was obtained. Table A in S1 File presents statistics characterizing the dataset.

### Evaluation metrics

To compare the prediction accuracy of the proposed approach, the precision, recall, F-measure, and MAP (Mean Average Precision), which are widely used in recommendation systems to evaluate the recommendation quality, were employed in this research [63]. Specifically, precision is the percentage of relevant papers out of those papers selected by the recommendation list while recall is the percentage of relevant papers selected out of all the relevant papers in the repository. F-measure considers the effect of both precision and recall at the same time and does not obscure any particular deficiencies; it can thus be used to evaluate the pros and cons of the algorithm more comprehensively. They are given as follows:

$$Precision@M=\frac{Number\;of\;relevant\;recommended\;papers\;in\;top\;M}{Totle\;Number\;of\;recommended\;papers\;M}$$ (15)

$$Recall@M=\frac{Number\;of\;relevant\;recommended\;papers\;in\;top\;M}{Totle\;Number\;of\;collected\;relevant\;papers\;of\;testing\;data}$$ (16)

F−measure@M=(2×Precision×Recall)(Precision+Recall) (17)

Where *M* is the number of recommendation list.

MAP assesses the overall performance based on precisions at different recall levels on a test dataset. It computes the mean of average precision (AP) over all researchers in the test dataset, where AP is the average of precisions computed at all positions with a preferred paper:

$$MAP@M=\frac{1}{U}\sum_{j=1}^{\left|U\right|}\frac{1}{m_{j}}\sum_{k=1}^{M}P\left(R_{j,k}\right)$$ (18)

Where |*U*| denotes the number of researchers, *m*_*j*_ is the number of relevant papers to the researcher *u*_*j*_, and *P*(*R*_*j*,*k*_) represent the precision of recommended results from the top result until reaching paper *v*_*k*_. *MAP*@*M* considers the rank information of relevant papers in the recommendation list.

### Experimental procedure

For the performance comparison in this research, some existing state-of-the-art paper recommendation approaches in the literature were implemented. They are listed as follows:

1. Probabilistic Matrix Factorization, i.e., PMF: We compare our method with the baseline method PMF proposed by Salakhutdinov et al. [64], which no negative instances are extracted, that is to say All Missing As Unknown (AMAU) [17, 65] without consideration of any social information. An ordered list of papers can be obtained by sorting the scores calculated by multiplying two low-rank matrices.
2. ldNMF: This method is Low-Density Non-negative Matrix Factorizations method which is the stat-of-the-art OCCF method represented by Sindhwani et al. [47]. We have reviewed it in Related work.
3. Tag_PMF: This method is proposed by Ma et al. [51], which no negative instances are extracted, that is to say AMAU, and the social tag information is used in the probabilistic matrix factorization instead of the social network in the original paper.
4. Friend_PMF [50]: No negative instances are extracted, that is to say AMAU, and only social friend information are used in the probabilistic matrix factorization.
5. PMF_AMAN [17]: All Missing as Negative. Neither social tag information nor social friend information are used in the probabilistic matrix factorization.
6. PMF_RMAN: Random Missing as Negative. Neither social tag information nor social friend information is used in the probabilistic matrix factorization.
7. PMF_DMAN: Dissimilar Missing as Negative. Through the process proposed by our approach, extract the papers that are dissimilar to that researcher’s preference as negative instances. Neither social tag information nor social friend information are used in the probabilistic matrix factorization.
8. OCCF_AMAN [17]: With AMAN strategy. Both social tag and social friend information are adopted in the probabilistic matrix factorization to enhance the recommendation performance.
9. OCCF _RMAN: With RMAN strategy. Both social tag and social friend information are adopted to conduct unified probabilistic matrix factorization.
10. OCCF_AMAU [65]: All Missing as Unknown. Use only positive instances with both social tag and social friend information to conduct unified matrix factorization.

The collected data were used to construct a researcher-paper matrix and randomly divided into training and testing datasets. 80% of researcher-paper matrix was used as the training dataset, and the remaining data were used as the test dataset. Since each column in the matrix corresponds to papers, the set of papers in test dataset was disjoint from those in the training dataset. To ensure the experimental results were not sensitive to the division of each dataset, all results reported here are averaged over 10 rounds, and each time we used different random splits. The hyper-parameters for all approaches were optimized via a grid search in the first round and kept constant in the remaining 9 rounds. The parameter settings of our approach were *θ*_*U*_ = *θ*_*V*_ = 1, *numFactors* = 10, and *numIter* = 100. In our experiments, the parameters of all algorithms were optimized for their best performance.

## Results and discussion

### Experimental results and analysis

In this section, the values for evaluation metrics were obtained and compared among the state-of-the-art approaches and our proposed approach applied to the CiteULike dataset. The detailed results are presented in Table B in S1 File. Table B in S1 File lists the performance in terms of evaluation metric for top @M (*M* = 10, 20, 30, 40, 50) obtained by the proposed hybrid SCORP and other compared approaches. The best results are marked in bold and underlined.

Generally, as indicated in Table B in S1 File, the hybrid SCORP outperforms the compared approaches with significant margins in all the metrics when recommending 10, 20, 30, 40 or 50 papers. The best performance in terms of the F-measure is achieved by our proposed hybrid SCORP method to 0.03341 at a recommendation number of @10 with an improvement of more than 19% on average. For MAP, the SCORP also obtains the highest performance, with a value of 0.09035. The experiments also reveal a number of interesting observations. For example, among these baseline approaches, the performance of PMF_DMAN, which does not use social information in the stage of probabilistic matrix factorization but rather extracts negative instances based on the similarity with social information, is always the best, even compared with ldNMF. In contrast, the PMF_RMAN, which randomly extract the negative instances from the unlabelled data, obtains the worst result in terms of precision, recall and the F-measure. This result demonstrates the effectiveness of social information as additive evidence to the baseline approaches. Mining social information to determine the recommended papers, the proposed hybrid SCORP is able to obtain the best results.

#### Results analysis of negative instance extraction with social information

**Fig 5:**
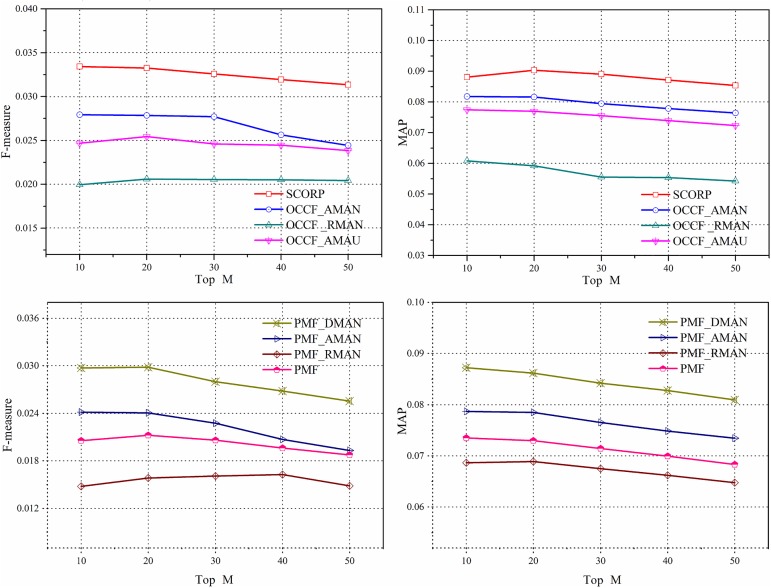
The influence of social information on negative instance extraction.

As mentioned above, the imbalance and sparsity problems are the two main barriers of OCCF for paper recommendation. Therefore, in this section, the effect of the negative instance extraction with social information is evaluated by comparing its recommendation quality to those of other extraction strategies. The compared results are shown in Fig 5. It can be easily observed from the figure that the two approaches with our proposed extraction strategy, the SCORP and the PMF_DMAN, achieved higher performance for both F-measure and MAP over the compared approaches. This result shows that social tags, as additional information, play a significant role in the paper recommendation when determine whether to extract a paper as a negative instance since the proposed hybrid SCORP achieves a performance improvement of more than 17% relative to the best competitive approach. These results imply the natural advantage of social tag information in characterization of researcher preference as well as paper feature. Specially, we can see that compared to the AMAU strategy, the AMAN strategy is more effective. This is just consisted with the conclusion in [17] that although the label information of unlabelled instances is unknown, we still have the prior knowledge that most of them are negative instances. Disregarding such information and using only positive instances does not lead to competitive recommendations. In addition to that, through a further observation, compared to the RMAN strategy, which randomly extract negative instances, the methods without extraction, namely, OCCF_AMAU and PMF, are even better. This can be explained by the fact that with RMAN, many potential positive instances were misclassified into negative instances. As a result, it damaged the training of the recommendation models instead. The above result proves the importance of mining social information to find credible and trustworthy negative instances.

#### Results analysis of OCCF prediction model with social information

**Fig 6:**
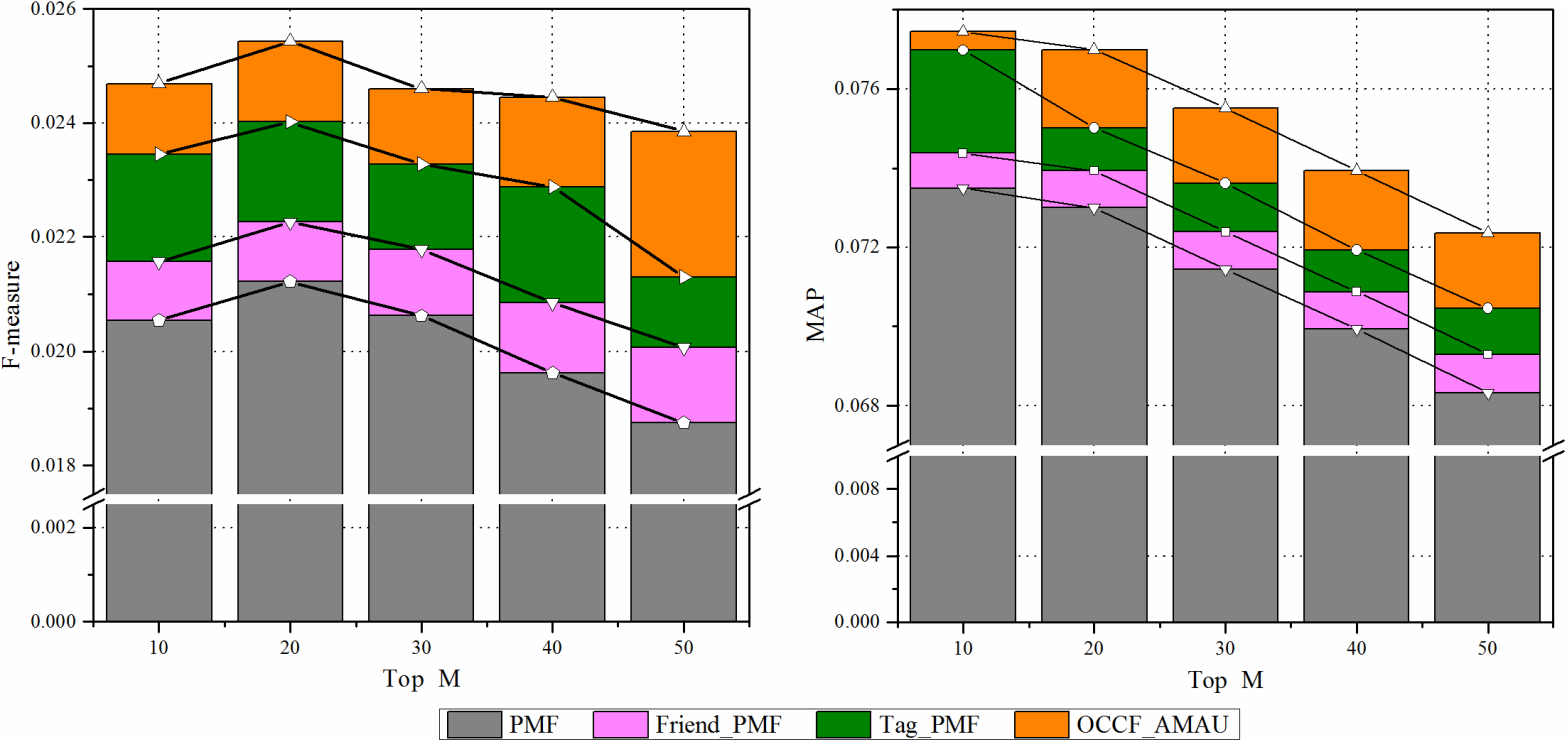
The influence of social information on prediction.

This section investigates the effect of different social information on the performance of paper recommendation in SSNs. We focus on the social tag and friend information of the CiteULike dataset due to their generality. Fig 6 depicts the performance improvement of them against the baseline PMF, which does not use any additional information in the stage of probabilistic matrix factorization. It is clear that the proposed hybrid SCORP outperforms in terms of the two performance metrics to a great extent compared with the other three benchmark approaches, which proves the effectiveness of social information in helping overcome the matrix sparsity. Specifically, according to the results presented in Fig 6, the social tag has a higher impact on the recommendation performance, which is attributed to the existence of domain relativity of the papers in SSNs. In SSNs, people’s preferences are more professional and thus more sensitive to the internal research topics of papers, which are refined as tags. Further, by integrating both the social tag and friend information, the hybrid SCORP increases the performance significantly. This is not merely because of their respective effectiveness but also benefits from the potential promoting interactions between them adaptively.

### Parameter discussion

For the proposed hybrid SCORP, the recommendation number *M*, the negative instance rate *β*, and the regularization parameters of *regularUserSim*, *regularItemTag*, *regularBiaoQian* are all important influence factors of the recommendation result. Therefore, further exploration of the recommendation performance under different parameters settings is conducted. All of these following experiments were performed on the CiteULike dataset, and all the values of MAP were obtained under the parameters settings at which the F-measure took the optimal value.

#### Discussion of the recommendation number *M*

**Fig 7:**
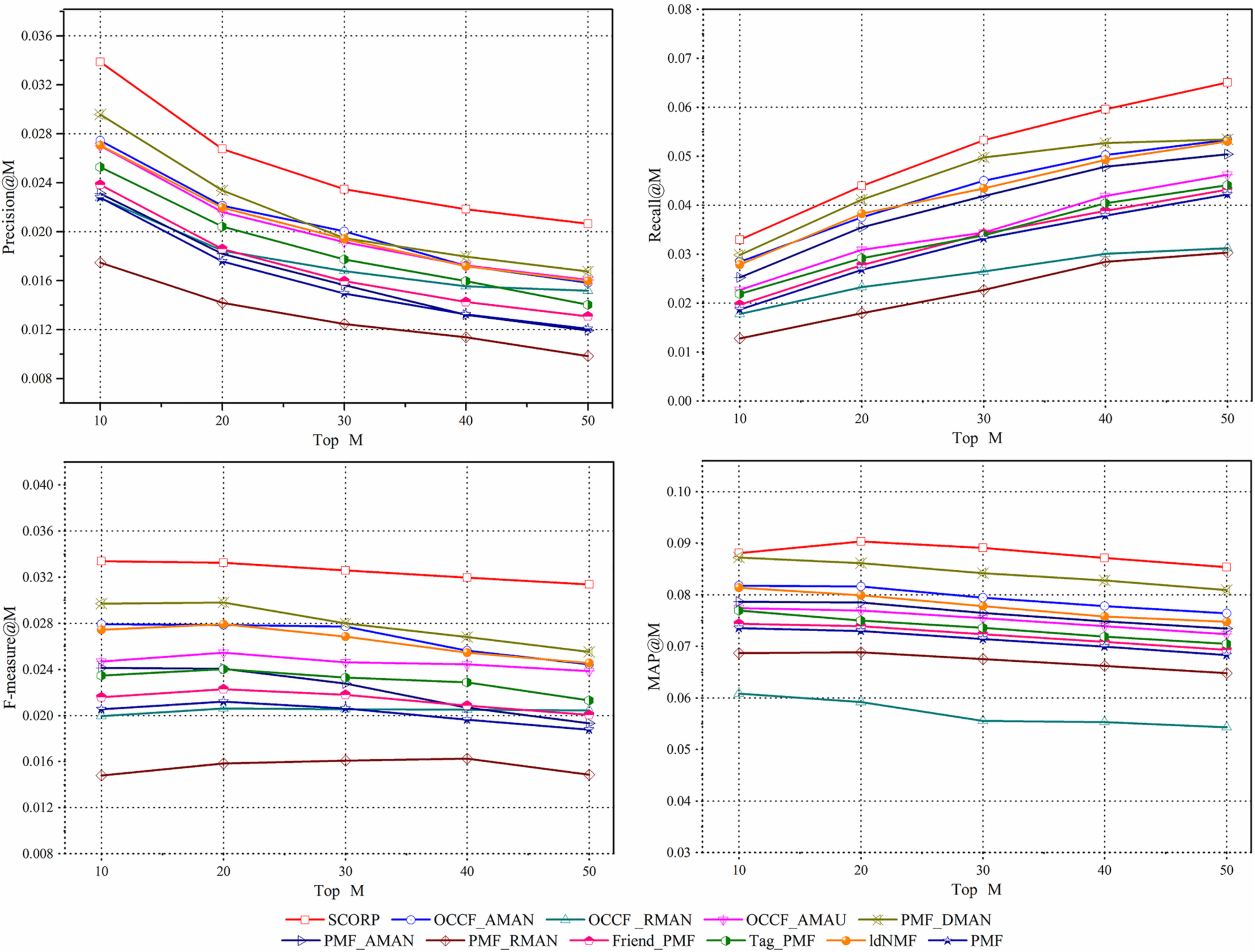
The influence of recommendation number *M* on proposed SCORP and other compared approaches.

For the personalized recommendation systems, the ultimate goal is not to make the error between the predicted ratings and the real ratings as small as possible but rather to recommend the most suitable list for the specific target user’s preference. Therefore, determining the optimal value of the recommendation number *M* is of great significance. It is evident from Fig 7 that with increased *M*, the precision is reduced significantly while the recall is increased to a certain degree. Furthermore, the F-measure, as an overall consideration of these two metrics, its values of the proposed SCORP decline slightly with increased *M*, it still far better than that of other methods. On the other hand, the stable outperforming state of the proposed SCORP in spite of the recommendation number also indicates the universality of it, whereas for the MAP, the influence of *M* on different methods seems very alike with a trend of a slight decline on the whole.

#### Discussion of the negative instance rate *β*

**Fig 8:**
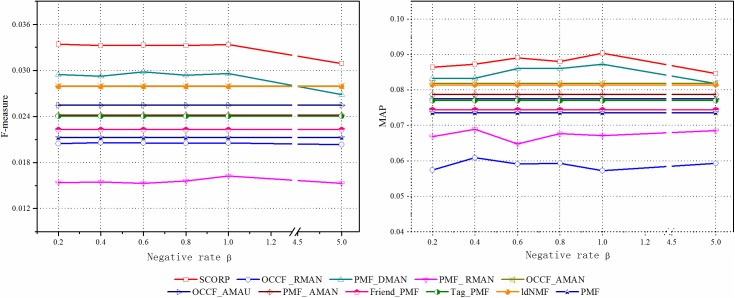
The influence of negative instance rate *β* on proposed SCORP and other compared approaches.

As described in Section Negative instance extraction with social information, the negative instance rate *β* is an important factor in the negative instance extraction. Fig 8 shows the F-measure and MAP of the proposed SCORP and compared approaches under different *β*. When the negative instance rate *β* becomes higher, the F-measure of the SCORP gradually declines on the whole except for the small bulge when *β* = 1.Our explanation is that a smaller negative instance rate indicates a better balance between positive and negative instances and a higher accuracy of the selected negative instances. As a result, together with these extracted negative instances, the original positive instances can express the researchers’ preferences more accurately. With increased *β*, on the one hand, increased noise is extracted unavoidably and causes some interference with the recommendation. On the other hand, too many negative instances will also affect the distribution of the data, resulting in the training results tending to be negative. An extreme instance can be observed from OCCF_AMAN, which regards all unlabelled as negative. Obviously, the performance of it was worse whether on F-measure or on MAP than the proposed SCORP from Fig 8, which is consistent with our explanation. For the small bulge, it is most likely due to the parameter tuning on other parameters (e.g., *regularItemTag*, *regularBiaoQian*, *regularUserSim*) towards the optimal accuracy when *β* = 1 as the default. Because the increasing *β* does not seem to incur a significant change in the recommendation when *β* ∈ [0.2,1]. Moreover, as *β* increases, the number of training data is largely increase, which leads to a very large computational overhead. We set *β* = 0.2 as default to save computational and storage costs.

#### Discussion of the parameter of *regularItemTag* and *regularBiaoQian*

**Fig 9:**
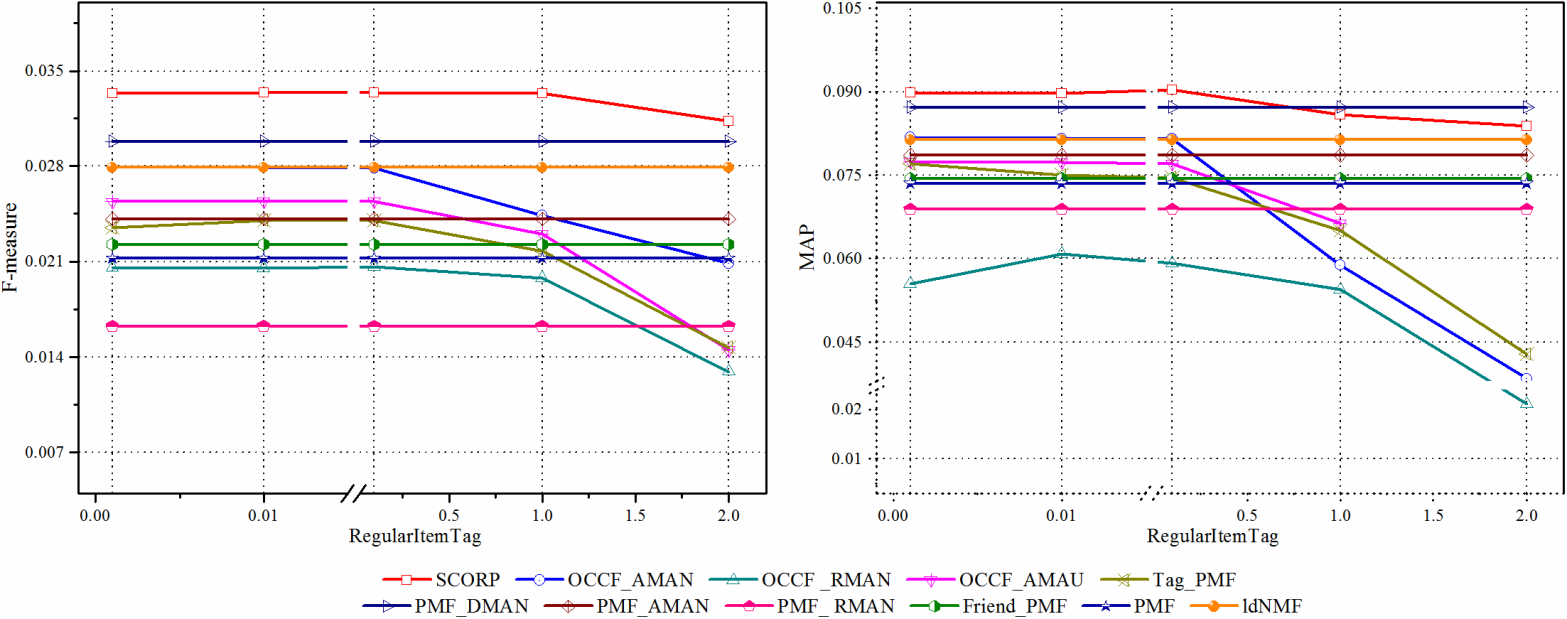
The influence of *regularItemTag* on proposed SCORP and other compared approaches.

**Fig 10:**
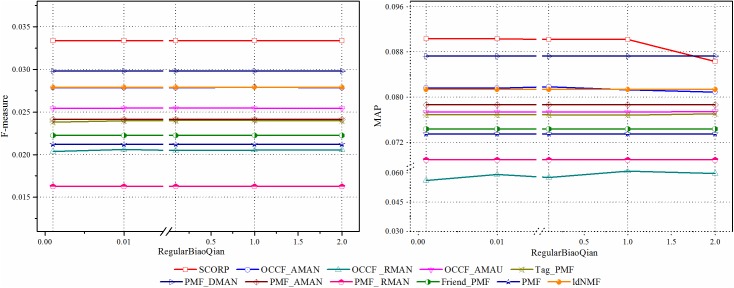
The influence of *regularBiaoQian* on proposed SCORP and other compared approaches.

One of the main advantages of the proposed SCORP is that it exploits the social information in SSNs. In our model, the parameters *regularItemTag* and *regularBiaoQian* balance the information from the researcher-paper matrix *R*^*M*×*N*^ and the paper-tag matrix *T*^*N*×*H*^. If *regularItemTag* = 0 and *regularBiaoQian* = 0, only the researcher-paper matrix *R*^*M*×*N*^ is used for probabilistic matrix factorization. Otherwise, if *regularItemTag* = inf or *regularBiaoQian* = inf, only the paper-tag matrix *T*^*N*×*H*^ is extracted for prediction. Figs 9 and 10 show the impact of *regularItemTag* and *regularBiaoQian* on F-measure and MAP. For *regularItemTag*, it is observed from Fig 9 that the value of the *regularItemTag* impacts the recommendation results significantly, which demonstrates that integrating the researcher-paper matrix with the social tag information greatly improves the recommendation accuracy. As *regularItemTag* increases, the recommendation F-measure and MAP increase at first, but when *regularItemTag* surpasses a certain threshold, the recommendation performance starts to decrease with a further increase of *regularItemTag*. The existence of the yielding point confirms with the intuition that purely using the researcher-paper matrix or purely using the paper-tag matrix *T*^*N*×*H*^ cannot generate better performance than appropriately integrating these two types of information together. For the CiteULike dataset in this study, the proposed SCORP achieves its best performance when *regularItemTag* is approximately 0.1. A similar change appears for *regularBiaoQian*, which has its highest F-measure and MAP at the value of 1. Compared with the *regularItemTag*, the results change of the *regularBiaoQian* is more insensitive, which shows that the parameter *regularItemTag* of our approach is easy to train to some extent.

#### Discussion of the parameter of *regularUserSim*

**Fig 11:**
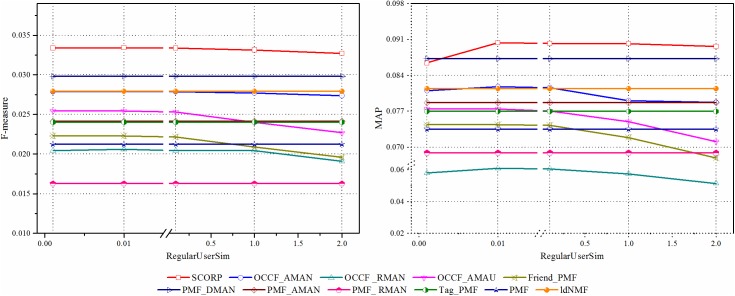
The influence of *regularUserSim* on proposed SCORP and other compared approaches.

As mentioned in the previous section, in the proposed SCORP, the parameter *regularUserSim* also plays a very important role. It controls how much the proposed approach should incorporate the information of the social friend in SSNs. Too large or too small values for *regularUserSim* will result in a bad recommendation. In many cases, we do not want to set *regularUserSim* to these extreme values since they will potentially hurt the recommendation performance. In this section, how the changes of *regularUserSim* can affect the final recommendation is analysed. Fig 11 shows the impacts of *regularUserSim* on F-measure and MAP. It can be observed that when the *regularUserSim* = 0.1, both the F-measure and MAP achieve their maximum. The impact of *regularUserSim* generally shares the similar trend as the impact of *regularItemTag* in Fig 9. Hence, no more repetition is performed here due to space limitations. However, it is worth noting that compared with the *regularItemTag*, the value of *regularUserSim* has less influence on the recommendation in CiteULike. The values of both *F-measure*@10 and *MAP*@10 barely change with the variations in *regularUserSim* values. This result also corresponds with the inference explained in Section Results analysis of OCCF prediction model with social information that papers in SSNs is domain relativity; therefore, recommendation is more sensitive to the impact of social tags labelled on them.

## Conclusions and future work

In this study, a hybrid approach of Social and Content aware One-class Recommendation of Papers in SSNs, termed SCORP is proposed. Social tag information was used in the negative instance extraction for profiling in the CBF, and then combining with the observed static statistics, the problem of extreme imbalance and sparsity can alleviated. Subsequently, when modelling and predicting, the social information in the SSNs, such as tag and friend information, is also embedded into the standard CF to conduct unified probabilistic matrix factorization, a further relieving of data sparsity of OCCF was made. The experimental results on real SSNs dataset, CiteULike, demonstrated that the proposed hybrid SCORP approach is superior to the compared recommendation approaches. With the proposed superior recommendation approach, researchers will benefit from time saved on web browsing, increased access to valuable information that they need and greater awareness up-to-date developments in their field.

There are several promising directions for future research. First, according to the results obtained, it is clear that additional social information contained in SSNs can greatly improve the recommendation accuracy. Therefore, in the future, besides studying on the treatment of fake noisy social information, this work will be extended by considering other available information, such as the time factor and the quality of the scientific papers (as quantified by citations and journal impact factor). Second, to address the extreme imbalance and sparsity problem inherent in the paper recommendation OCCF problem, more complex methods, such as incremental iterative updating, can be implemented to produce better results. Third, in this study, only one type of SSNs, CiteUlike, was employed to verify the proposed hybrid SCORP approach. In the future, we will extend our approach to other platforms, such as Research Gate, ScholarMate, and Academia.edu. And further explore the combination of heterogeneous data sources to improve the recommendation applicability and generality.

## Supporting information

S1 Data CiteULikeData-Paper-User-Tag-Data1. (RAR)

S2 Data CiteULikeData-Paper-User-Tag-Data2. (RAR)

S3 Data CiteULikeData-Paper-User-Tag-Data3. (RAR)

S4 Data CiteULikeData-User-Friends-Data. (RAR)

S1 File **Table A Statistics of filtered CiteULike dataset and Table B Precision recall, F-measure and MAP results for hybrid SCORP and other compared approaches**.
(RAR)
